# Neuronal Cells Display Distinct Stability Controls of Alternative Polyadenylation mRNA Isoforms, Long Non-Coding RNAs, and Mitochondrial RNAs

**DOI:** 10.3389/fgene.2022.840369

**Published:** 2022-05-18

**Authors:** Aysegul Guvenek, Jihae Shin, Lidia De Filippis, Dinghai Zheng, Wei Wang, Zhiping P. Pang, Bin Tian

**Affiliations:** ^1^ Department of Microbiology, Biochemistry and Molecular Genetics, Rutgers New Jersey Medical School, Newark, NJ, United States; ^2^ Rutgers School of Graduate Studies, Newark, NJ, United States; ^3^ Department of Neuroscience and Cell Biology, Child Health Institute of New Jersey, Rutgers Robert Wood Johnson Medical School, New Brunswick, NJ, United States; ^4^ Program in Gene Expression and Regulation, Center for Systems and Computational Biology, The Wistar Institute, Philadelphia, PA, United States

**Keywords:** alternative polyadenylation, 3′UTR, RNA stability, mitochondrial RNA, long non-coding RNA, GC content, RNA structure

## Abstract

RNA stability plays an important role in gene expression. Here, using 3′ end sequencing of newly made and pre-existing poly(A)+ RNAs, we compare transcript stability in multiple human cell lines, including HEK293T, HepG2, and SH-SY5Y. We show that while mRNA stability is generally conserved across the cell lines, specific transcripts having a high GC content and possibly more stable secondary RNA structures are relatively more stable in SH-SY5Y cells compared to the other 2 cell lines. These features also differentiate stability levels of alternative polyadenylation (APA) 3′UTR isoforms in a cell type-specific manner. Using differentiation of a neural stem cell line as a model, we show that mRNA stability difference could contribute to gene expression changes in neurogenesis and confirm the neuronal identity of SH-SY5Y cells at both gene expression and APA levels. In addition, compared to transcripts using 3′-most exon cleavage/polyadenylation sites (PASs), those using intronic PASs are generally less stable, especially when the PAS-containing intron is large and has a strong 5′ splice site, suggesting that intronic polyadenylation mostly plays a negative role in gene expression. Interestingly, the differential mRNA stability among APA isoforms appears to buffer PAS choice in these cell lines. Moreover, we found that several other poly(A)+ RNA species, including promoter-associated long noncoding RNAs and transcripts encoded by the mitochondrial genome, are more stable in SH-SY5Y cells than the other 2 cell lines, further highlighting distinct RNA metabolism in neuronal cells. Together, our results indicate that distinct RNA stability control in neuronal cells may contribute to the gene expression and APA programs that define their cell identity.

## 1 Introduction

Regulation of RNA stability is an important step of expression control of both protein-coding and noncoding genes (Garneau et al., 2007; Schoenberg and Maquat, 2012; Kilchert et al., 2016; Schmid and Jensen, 2019). Distinct decay mechanisms exist in cytoplasm and nucleus, responsible for modulating the life spans of cytoplasm-enriched mRNAs and nucleus-enriched long non-coding RNAs (lncRNAs), respectively (Garneau et al., 2007; Schoenberg and Maquat, 2012; Kilchert et al., 2016; Schmid and Jensen, 2019). RNA decay regulation is also critical for removing aberrant RNA species in the cell (Wolin and Maquat, 2019). Many of the mRNA decay mechanisms are modulated by 3′UTR sequence motifs, such as AU-rich elements (AREs) and GU-rich elements (GREs) (Chen and Shyu, 1995; Barreau et al., 2005; Vlasova et al., 2008; Lee et al., 2010; Vlasova-St Louis et al., 2013), through interactions with a large repertoire of RNA binding proteins (RBPs) (Hentze et al., 2018). In addition, structured RNAs in 3′UTRs could regulate mRNA decay by interacting with RBPs that have direct roles in RNA stability (Park and Maquat, 2013; Fischer et al., 2020) or by influencing decay kinetics (Wu and Bartel, 2017). Moreover, 3′UTR size *per se* has also been reported as a destabilizing feature due to 3′UTR size-dependent interaction with UPF1 (Hogg and Goff, 2010).

Cleavage/polyadenylation of RNA precursors defines the 3’ end of almost all mRNAs and lncRNAs. About 70% of mRNA genes in mammals have multiple cleavage/polyadenylation sites (PASs) leading to mRNA isoforms, a phenomenon called alternative polyadenylation (APA) (Tian and Manley, 2017; Gruber and Zavolan, 2019). APA is dynamic in cell growth, differentiation and development (Sandberg et al., 2008; Ji et al., 2009; Shepard et al., 2011). Different tissue or cell types display distinct global APA profiles (Zhang et al., 2005). For example, neuronal cells prefer the usage of APA sites that are distal to gene promoter compared to other cell types (Zhang et al., 2005; Miura et al., 2013; Guvenek and Tian, 2018). In contrast, immune cells and professional secretory cells globally prefer proximal APA sites (Singh et al., 2018; Cheng et al., 2020). Genes that display APA tend to be ubiquitously expressed (Lianoglou et al., 2013) and have long evolutionary history (Lee et al., 2008; Wang et al., 2018b). Notably, brain-specific genes were found to have a higher APA site conservation rate than other genes (Wang et al., 2018b), suggesting that APA isoform expression may be particularly important in neuronal cells.

APA sites in the 3′-most exon lead mostly to isoforms with different 3′UTR sizes, whereas APA sites in introns change both the coding region and 3′UTR. Because of the central role of 3′UTR in mRNA metabolism (Mayr, 2018), APA isoforms with different 3′UTR sizes are expected to have distinct fates in the cell. Indeed, a growing number of studies have shown that 3′UTR isoforms can differ substantially in stability, translational efficiency, or localization (Spies et al., 2013; Tushev et al., 2018). For example, long 3′UTR isoforms in general are less stable than short 3′UTR isoforms in mouse NIH3T3 cells (Spies et al., 2013; Zheng et al., 2018) and human HEK293T cells (Shin et al., 2021). However, to what extent stability difference between isoforms varies across cell types remains an open question.

In this study, by comparing poly(A)+ transcript abundance in newly made and pre-existing pools, we examine how APA isoforms differ in stability in multiple human cell lines. We examine how stability difference between isoforms varies across cell types and analyze the interplay between stability control and APA site choice. We further examine stability controls of lncRNAs and poly(A)+ RNAs encoded by the mitochondrial genome. Our results reveal 3′UTR features correlated with transcript stability differences between cell types and indicate potential contributions of RNA stability to neuronal cell identity.

## 2 Results

### 2.1 Global Stability Analysis of poly(A)+ RNAs in Three Human Cell Lines

**FIGURE 1:**
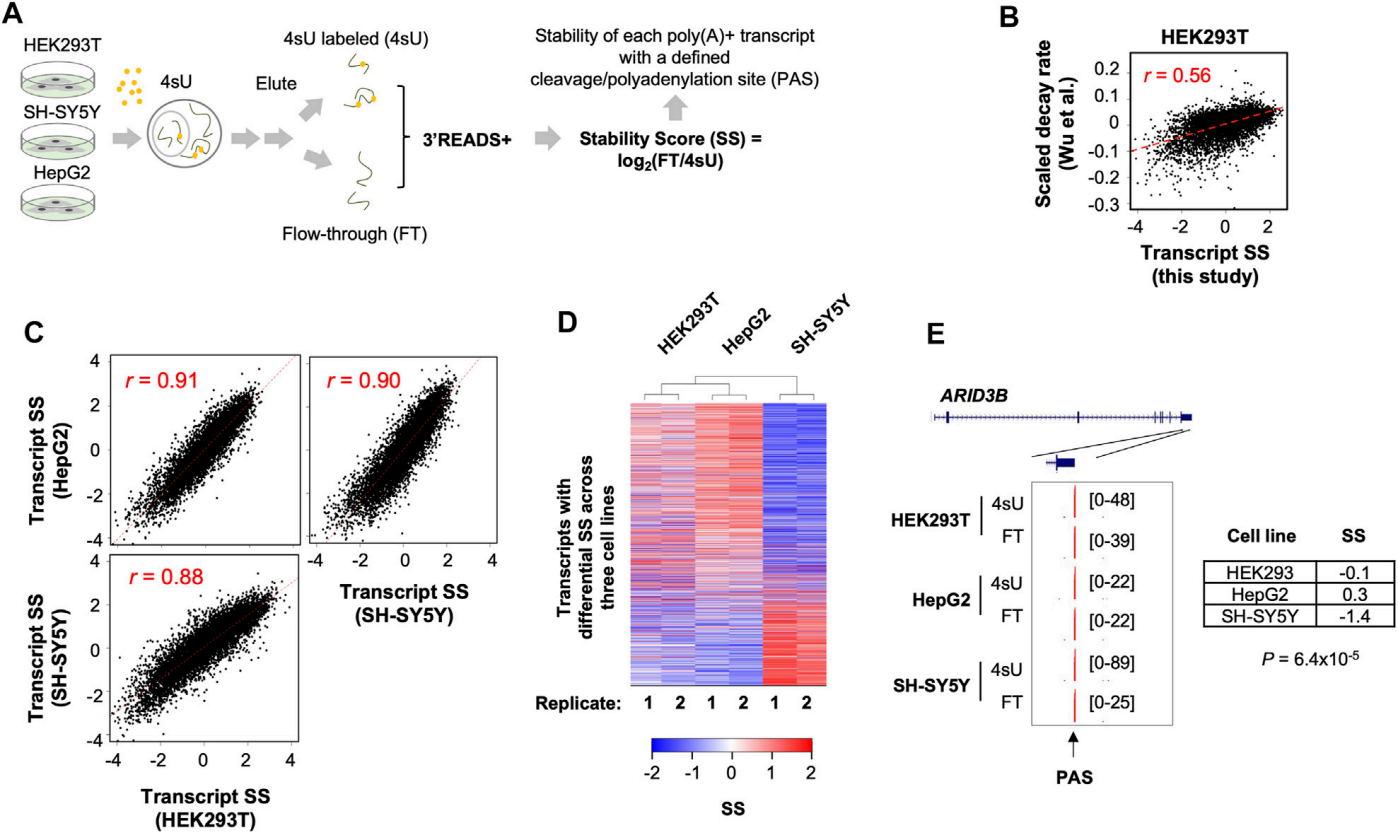
Systemic analysis of poly(A)+ RNA stability in three human cell lines. **(A)** Schematic of experimental procedure. HEK293T, HepG2, and SH-SY5Y cells were subject to metabolic labeling with 4-thiouridine (4sU) for 1 h. Total cellular RNA was extracted from each sample and was fractionated into 4sU-labeled (4sU) and unlabeled (flow-through or FT) fractions. Isolated RNA was subject to 3′READS+ for 3′ end sequencing. Stability Score (SS) of each transcript (represented by its polyA site, or PAS) was calculated by log_2_(FT/4sU), where FT and 4sU are transcript abundances in the FT and 4sU fractions, respectively. **(B)** Comparison of SS of transcripts in HEK293T cells (this study) with scaled decay rates from a previous study by Wu et al. (GSE126520, NCBI GEO). Pearson correlation coefficient *r* is indicated. **(C)** Pair-wise comparisons of mRNA SS between 3 cell lines. Pearson correlation coefficient *r* is indicated. Each dot is a gene. When a gene has multiple PAS isoforms, the one with the highest expression value based on the average of all 3 cell lines is used. **(D)** Heatmap showing mRNA genes with differential SS among the 3 cell lines. Each row is an mRNA gene. Two-way hierarchical clustering was based on Euclidian distance. Genes with significant differential stability across the cell lines are those with *p* < 0.05 (ANOVA test). **(E)** UCSC tracks showing an example gene *ARID3B*. SS in each cell line and *p*-value indicating significance of difference in SS (ANOVA test) are shown. PAS is indicated by an arrow.

We hypothesized that transcript stability might be distinct in different cell types. To test this, we carried out metabolic labeling of cellular RNA with 4-thiouridine (4sU) in human HEK293T, HepG2, and SH-SY5Y cells (Figure 1A). These cell lines, all widely used in biomedical research, have distinct characteristics: HEK293T was derived from human embryonic kidney cells (DuBridge et al., 1987); HepG2 was from a liver hepatocellular carcinoma (Knowles et al., 1980) and has been a cell model for metabolism studies; SH-SY5Y was from a neuroblastoma (Biedler et al., 1978) and has been a model to study neuronal cell functions (Kovalevich and Langford, 2013).

After 4sU labeling for 1 h, total cellular RNA was extracted from each cell line (two biological replicates each), followed by separation of 4sU-labeled and non-labeled RNAs (see Methods for details). These two RNA pools, named 4sU labeled (4sU) and flow-through (FT), respectively, represent newly made and pre-existing RNAs, respectively. We subjected 4sU and FT RNA samples, as well as the total RNA, to the 3′READS+ method for 3′ end sequencing (Jin et al., 2015; Zheng et al., 2016). The abundance of each transcript is represented by reads mapped to its PAS (Zheng et al., 2016). We calculated the ratio of transcript abundance in the FT pool to that in the 4sU pool, or log_2_(FT/4sU), named Stability Score (SS) for simplicity, to represent RNA stability (Zheng et al., 2018). We found that SS values of transcripts in HEK293T cells were well correlated (*r* = 0.56, Pearson Correlation, Figure 1B) with scaled decay rates measured by using transcriptional shutdown with Actinomycin D (Wu et al., 2019), supporting the validity of using SS to reflect transcript stability.

Requiring at least five reads in at least one sample as evidence of expression (Supplementary Figure S1A), we identified over 100,000 PASs in the 3 cell lines (Supplementary Figure S1A). We then used RefSeq and GENCODE databases to assign identified PASs to genes (Frankish et al., 2019). Unassigned reads were additionally annotated with the Fantom database, which has a good coverage of lncRNAs (Lizio et al., 2015; Hon et al., 2017), including promoter-associated lncRNA (p-lncRNA) and enhancer-associated RNAs (eRNAs). Overall, we identified PASs to over 15,000 mRNA and lncRNA genes (Supplementary Tables S1, S2), of which 11,555 were detected in all 3 cell types. Our gene types included mRNA, lncRNA, eRNA, p-lncRNA, pseudogene, mitochondrial genome gene, and intergenic transcripts (Supplementary Figure S1B). Interestingly, 4sU and FT samples showed distinct distributions for different gene types (Supplementary Figure S1B), suggesting different RNA species are grossly different in stability.

### 2.2 Consistent and Distinct mRNA Stability Across Cell Lines

We first focused on mRNA genes, which accounted for the majority of poly(A)+ RNAs (Supplementary Figure S1B). Overall, SS of mRNA transcripts were well correlated among the 3 cell lines (*r* = 0.88–0.91, Pearson Correlation, Figure 1C), indicating that mRNA stability is largely conserved across cell types. Gene Ontology (GO) analysis of the transcripts that were among the most stable (top 20% in SS) or the least stable (bottom 20% in SS) in all 3 cell lines (Supplementary Table S3) indicated that stable mRNAs were related to aspects of cell metabolism, such as “small molecule metabolic process”, “ion transport”, “oxidation-reduction”, and “NADH metabolic process”, whereas unstable mRNAs were related to gene expression regulation, such as “transcription from RNA polymerase II promoter”, “negative regulation of macromolecule biosynthetic process”, and “cell differentiation”. In addition, several well-known mRNA stability-related gene features were well correlated with SS (Supplementary Figure S2), such as GC content (both gene and last exon) and intron/exon junction density as positive features for mRNA stability and U content (both gene and 3′UTR) and 3′UTR size as negative features for mRNA stability. Both GO and gene feature analysis results are in line with previous studies of mRNA stability (Lee et al., 2010; Spies et al., 2013), further supporting the suitability of using SS to examine RNA stability.

While transcripts were generally correlated in SS between the 3 cell lines, some differences were discernable (Figure 1C). We thus employed an ANOVA analysis to specifically identify transcripts that displayed significant stability difference among the cell lines, yielding 1,607 transcripts with *p* < 0.05 (ANOVA test, Figure 1D). Two-way hierarchical clustering of transcript SS revealed that SH-SY5Y was distinct from HEK293T and HepG2 (Figure 1D). An example gene *ARID3B* is shown in Figure 1E, whose SS in SH-SY5Y cells was much lower than those in HEK293T and HepG2 (−1.4 vs. −0.1 and 0.3, *p* = 6.4 × 10^−5^, ANOVA test, Figure 1E). Together, these results indicate that while transcript stability is largely similar among cell types, some transcripts display cell type-specific stability.

### 2.3 Distinct Stability Control in SH-SY5Y Cells is Relevant to Neuronal Cell Identity

**FIGURE 2:**
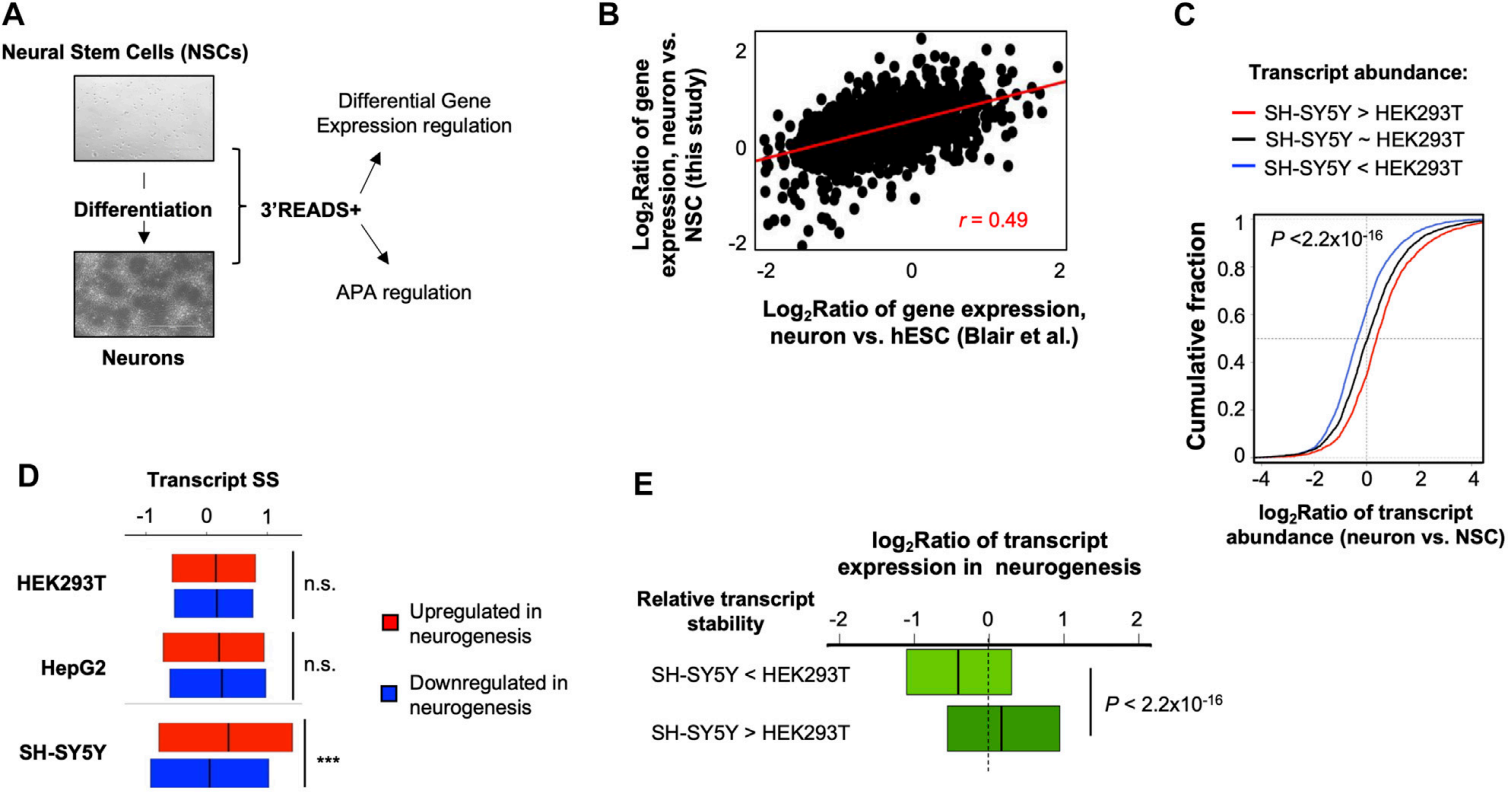
Distinct mRNA stability in SH-SY5Y cells is relevant to gene expression in neurogenesis. **(A)** A neurogenesis model used in this study. A neural stem cell (NSC) line (top) was differentiated to neurons (bottom), followed by 3′READS+ analysis by using their total cellular RNAs. The data were used for both gene expression and APA analyses. **(B)** Scatter plot showing gene expression changes between NSC-derived neurons vs. NSCs and another neurogenesis model, in which neurons were differentiated from human Embryonic Stem Cells (hESCs) (Blair et al.). **(C)** Cumulative distribution function (CDF) curves of transcript expression changes (log_2_Ratio) in NSC-derived neurons vs. NSCs for three groups of transcripts based on expression difference between SH-SY5Y and HEK293T cells, as indicated. Significance of expression difference of transcripts was based on Fisher’s exact test by using 3′READS+ data of total cellular RNA. The *p*-value (Wilcoxon test) shown in the plot indicates significance of difference between transcripts with higher expression in SH-SY5Y cells than in HEK293T cells (red line) and transcripts with higher expression in HEK293T cells than in SH-SY5Y cells (blue line). **(D)** SS of transcripts in 3 cell lines for genes upregulated or downregulated in neurogenesis. Comparison between the two transcript groups was based on Wilcoxon test. n.s., not significant; ***p < 0.001. **(E)** Box plot showing gene expression regulation in neurogenesis (Blair et al.) for two groups of genes with differential stability between SH-SY5Y and HEK293T cells, as indicated. Stability difference between SH-SY5Y and HEK293T was based on data shown in Figure 1C. *p*-value is based on Wilcoxon test.

Given the distinct mRNA stability profile in SH-SY5Y cells compared to HEK293 and HepG2 cells, we wondered whether mRNA stability might play a role in their neuronal cell identity. To this end, we carried out 3′READS+ analysis of a neural stem cell (NSC) line (Vescovi et al., 1999) and its differentiated neurons (Figure 2A, see Materials and Methods for detail). Consistent with the notion that differentiation of this NSC line provides a simple and meaningful analysis of neurogenesis (Vescovi et al., 1999), we found that gene expression changes in NSC versus derived neurons were well correlated with those in differentiation of human embryonic stem cells (hESCs) to mature neurons (Blair et al., 2017) (*r* = 0.49, Pearson Correlation, Figure 2B).

Based on total RNA samples, we found that the transcripts with a higher abundance in SH-SY5Y cells compared to HEK293T cells tended to be upregulated in neurogenesis, whereas those with a lower abundance in SH-SY5Y cells compared to HEK293T cells tended to be downregulated in neurogenesis (Figure 2C). This result is in line with the neuronal identity of SH-SY5Y cells and suggests that SH-SY5Y versus HEK293T comparison could provide insights into differences between neuronal and nonneuronal cells.

Interestingly, we found that genes upregulated in neurogenesis had higher SS than those downregulated in neurogenesis in SH-SY5Y cells (*p* < 0.001, Wilcoxon test, Figure 2D). By contrast, the SS difference between these two gene sets was not statistically significant in either HEK293T or HepG2 cells (Figure 2D). This result indicates that neuron-specific genes are more stable in SH-SY5Y cells than in nonneuronal cells. In line with this result, we found that transcripts tended to be upregulated in neurogenesis if they were more stable in SH-SY5Y cells than in HEK293T cells, and tended to be downregulated in neurogenesis if they were less stable in SH-SY5Y cells than in HEK293T cells (*p* < 2.2 × 10^−16^, K-S test, Figure 2E).

Gene Ontology analysis indicated that genes whose transcripts were more stable in SH-SY5Y cells than HEK293T cells tended to be associated with protein targeting to membrane and translation (Table 1), including “co-translational protein targeting to membrane”, “protein targeting to membrane”, “translational initiation”, etc. By contrast, genes whose transcripts were less stable in SH-SY5Y cells than in HEK293T cells tended to be associated with RNA processing and metabolism functions (Table 1), such as “RNA processing”, “ncRNA metabolic process”, “regulation of tolerance induction”, etc. Together, these results indicate that SH-SY5Y cells have distinct mRNA stability controls that impact specific functional gene groups and may play a role in establishing neuronal identity.

**TABLE 1:** Top biological processes enriched for transcripts with differential stability in SH-SY5Y cells versus HEK293T cells.

Biological process	*p*-value
Transcripts more stable in SH-SY5Y	
cotranslational protein targeting to membrane	1.8E-08
protein targeting to membrane	1.8E-08
translational initiation	2.5E-08
SRP-dependent cotranslational protein targeting to membrane	4.9E-08
protein localization to endoplasmic reticulum	1.6E-07
NADH regeneration	7.2E-04
canonical glycolysis	7.2E-04
monosaccharide catabolic process	1.9E-03
cofactor metabolic process	2.9E-03
hexose catabolic process	3.8E-03
Transcripts more stable in HEK293T	
RNA processing	7.2E-08
ncRNA metabolic process	5.5E-06
regulation of tolerance induction	6.7E-05
N-terminal peptidyl-methionine acetylation	6.7E-05
ncRNA processing	1.2E-04
S-adenosylmethionine metabolic process	2.6E-02
RNA polyadenylation	2.1E-02
cellular macromolecule catabolic process	2.1E-02
RNA 3′-end processing	4.2E-02
regulation of mRNA metabolic process	4.8E-02

### 2.4 Long 3′UTR Isoforms are Generally Less Stable than Short 3′UTR Isoforms

**FIGURE 3:**
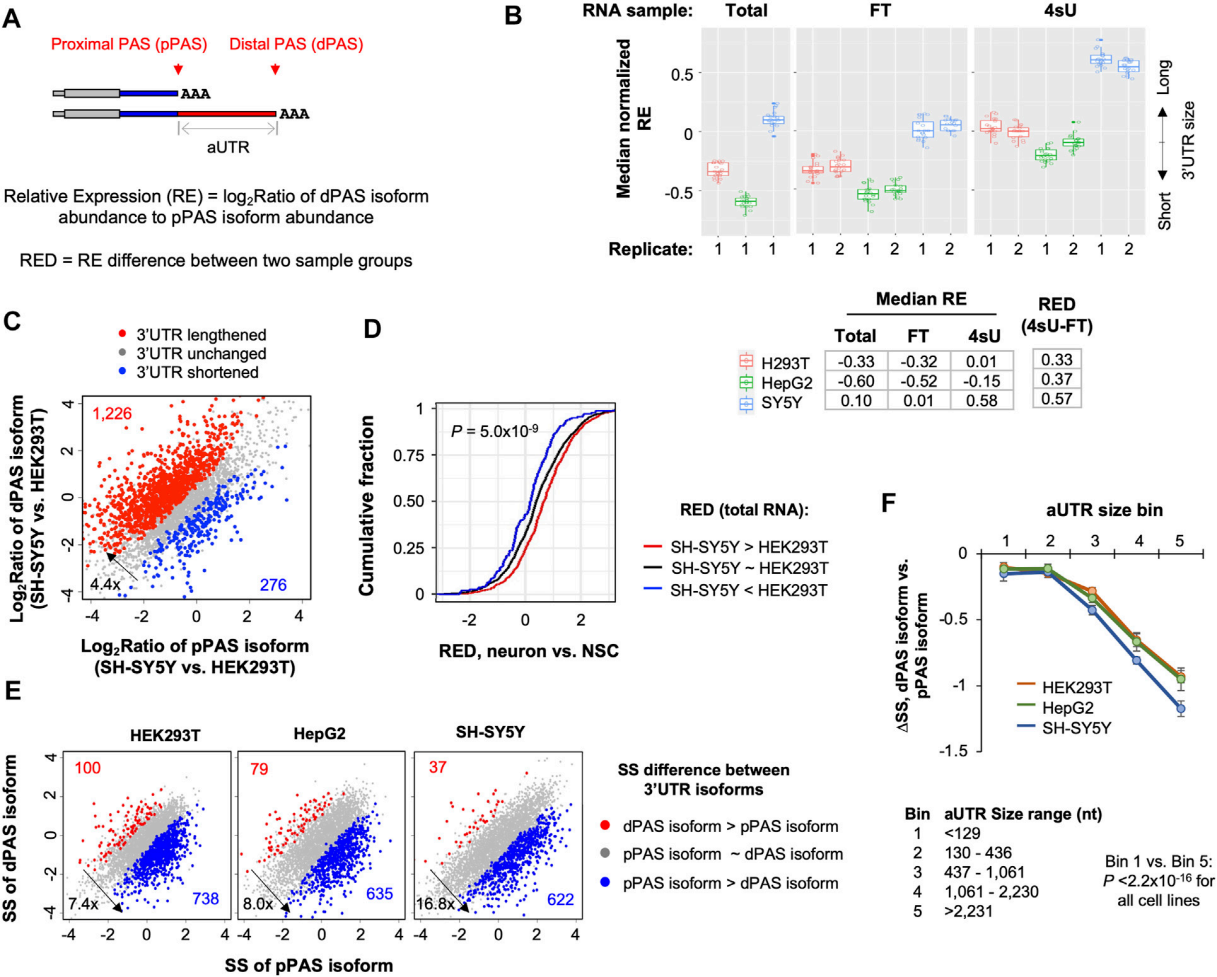
Long 3′UTR isoforms are generally less stable than short 3′UTR isoforms in all 3 cell lines. **(A)** Schematic showing two 3′UTR isoforms using proximal PAS (pPAS) or distal PAS (dPAS), respectively. The region between the two PASs is named alternative UTR (aUTR). Relative Expression (RE) of two isoforms is based on the abundance of dPAS isoform to that of pPAS isoform. RED is RE difference between two samples. **(B)** Box plot of median normalized RE values in total, FT and 4sU samples from 3 cell lines. The median value is based on all genes with 3′UTR isoform expression. The RE value of each gene in a cell line is normalized to the median RE value of all cell lines. Each box shows 20 median values based on bootstrapped data (*n* = 20). Median RE values for each sample is also shown in the table below the box plot. RED values between 4sU and FT samples are also shown. **(C)** 3′UTR isoform abundance difference between SH-SY5Y cells and HEK293T cells. The relative abundance of pPAS isoform and dPAS isoform indicates overall 3′UTR length. Genes whose 3′UTRs are longer in SH-SY5Y are shown in red, and those whose 3′UTRs are longer in HEK293T cells are in blue. **(D)** Cumulative distribution function (CDF) curves of 3′UTR APA RED values of differentiated neurons vs. NSCs for genes showing 3′UTR isoform abundance differences in SH-SY5Y vs. HEK293T cells [shown in **(C)**]. *p*-value (Wilcoxon test) for significance of difference between red and blue genes is indicated. **(E)** Scatter plots showing stability difference between short 3′UTR (pPAS) isoform and long 3′UTR (dPAS). Each dot is a gene with two selected 3′UTR isoforms. Genes whose dPAS isoform is more stable than pPAS isoform (FDR <0.05, DEXSeq) are in red and genes whose pPAS isoform is more stable than dPAS isoform in blue. The numbers of red and blue genes as well as their ratio of are indicated in each plot. **(F)** Difference in Stability Score (ΔSS) between dPAS isoform and pPAS isoform for genes with different aUTR sizes. Genes are divided into five equally sized bins based on their aUTR size. aUTR size range for each bin group is shown at the bottom. *p*-value (Wilcoxon test) for significance of difference between bin 1 and bin 5 is indicated.

APA in 3′UTR leads to isoforms with different 3′UTR sizes (Figure 3A). Previous studies have shown that 3′UTR isoforms could have different stability levels (Spies et al., 2013; Tushev et al., 2018; Zheng et al., 2018). We identified 3′UTR APA isoforms in 57% of all genes (6,568 out of 11,555) in the 3 cell lines. For simplicity, we focused on the top two 3′UTR APA isoforms based on the combined expression level of each isoform across the 3 cell lines. Based on the relative positions of their PASs to the 5′ end of gene, the two isoforms were named proximal PAS (pPAS) isoform and distal PAS (dPAS) isoform, respectively (Figure 3A); the 3′UTR portion between the two PASs was named alternative 3′UTR or aUTR (Figure 3A).

We calculated the relative expression (RE, log_2_Ratio of transcript abundance, illustrated in Figure 3A) of dPAS isoform versus pPAS isoform for each gene in each cell line (Figure 3B). Based on the median RE value of all genes, we found that, in both 4sU and FT samples, SH-SY5Y cells had much higher RE values than HepG2 and HEK293T cells (Figure 3B). This result is in good agreement with the notion that neuronal cells preferentially express long 3′UTRs compared to other cell types (Zhang et al., 2005; Miura et al., 2013; Guvenek and Tian, 2018; Ha et al., 2018). HepG2 cells had slightly lower RE values than HEK293T cells (Figure 3B), indicating that the former had the shortest 3′UTRs overall among the 3 cell lines.

We next directly compared SH-SY5Y cells with HEK293T cells for 3′UTR isoform expression levels (Figure 3C). We found that genes showing longer 3′UTRs in SH-SY5Y cells than HEK293T cells outnumbered those showing the opposite trend by 4.4-fold (red dots vs. blue dots, Figure 3C). Importantly, genes showing higher RE values (red dots in Figure 3C) in SH-SY5Y cells also tended to have higher RE values in neurogenesis (neurons versus NSCs, Figure 3D and Supplementary Figure S3A). Notably, both SH-SY5Y versus HEK293T and neurons versus NSCs showed aUTR size-dependent RE Difference (RED) increase (Supplementary Figure S3B), indicating that the larger the aUTR the more likely there is a switch from pPAS usage to dPAS usage, or 3′UTR lengthening. An example gene *ERCC1* is shown in Supplementary Figure S3C. These results indicate that comparison of SH-SY5Y with HEK293T recapitulates 3′UTR APA isoform changes in neurogenesis, both in terms of direction and degree.

Interestingly, we found that, in all cell lines, RE values were higher in 4sU samples than in FT samples (Figure 3B), indicating that 3′UTRs are longer in newly made RNAs than in pre-existing RNAs. As expected, RE values were similar between total and FT samples, indicating that pre-existing RNA (FT) is similar to steady state RNA (total). This result suggests that 3′UTR isoforms have distinct stability levels, leading to abundance differences in newly made versus pre-existing or steady state pools.

To examine 3′UTR isoform stability difference directly, we compared pPAS isoforms with dPAS isoforms for their SS in the 3 cell lines. Consistent with our previous data with mouse NIH3T3 cells (Zheng et al., 2018), we found that, in all these human cell lines, pPAS isoforms were significantly more stable than dPAS isoforms (*p* < 0.05, DEXSeq, Figure 3E). The number of genes whose pPAS isoforms were more stable than dPAS isoforms (blue genes in Figure 3E) outnumbered those with the opposite trend (red genes in Figure 3E) by 7.4-, 8.0-, and 16.8-fold in HEK293T, HepG2, and SH-SY5Y cells, respectively. The larger fold difference in SH-SY5Y cells than those in other 2 cell lines indicates that the extent to which long 3′UTR isoforms are less stable than short 3′UTR isoforms is the greatest in SH-SY5Y cells. We also found that as aUTR size increased, the difference in abundance between pPAS and dPAS isoforms became larger (Figure 3F). This trend appeared more obvious in SH-SY5Y cells than in HEK293T or HepG2 cells, further indicating that stability difference between 3′UTR isoforms is greater in SH-SY5Y cells compared to HEK293T and HepG2 cells.

### 2.5 3′UTR Motifs and Structures Contribute to Distinct mRNA Stability Controls in SH-SY5Y Cells

**FIGURE 4:**
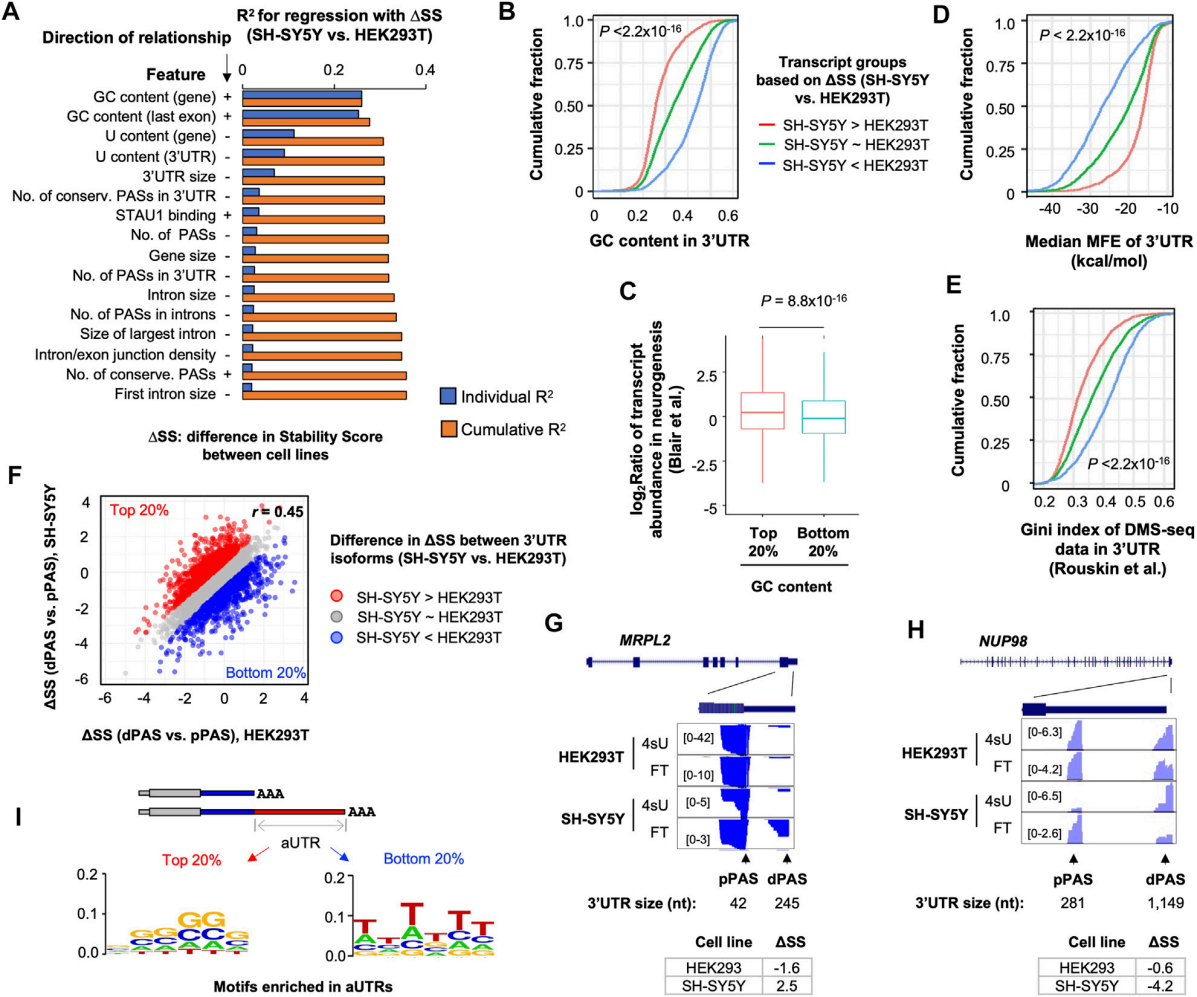
GC content and RNA secondary structures contribute to distinct mRNA stability in SH-SY5Y cells. **(A)** Top features associated with Stability Score difference (ΔSS) between SH-SY5Y cells and HEK293T, based on a linear regression analysis. Features are sorted according to individual *R* ^*2*^ values. The cumulative *R* ^2^ value is based on a given feature combined with all other features with a better individual *R* ^*2*^ value. Direction of relationship is indicated by + and –, denoting positive and negative correlations, respectively. **(B)** Cumulative distribution function (CDF) curves comparing 3′UTR GC content of three transcript groups, based on ΔSS between SH-SY5Y and HEK293T cells. *p*-value (Wilcoxon test) indicating significance of difference between red and blue genes is indicated. **(C)** Box plot showing gene expression regulation in neurogenesis (neurons vs. hESCs, Blair et al.) for genes with high (top 20%) or low (bottom 20%) 3′UTR GC contents. **(D)** As in B, except that median minimum folding energy (MFE) of 3′UTR is plotted. **(E)** As in B, except that RNA structure probing data from DMS-Seq are shown. Gini index reflects likelihood of RNA structures. **(F)** Scatter plot comparing ΔSS (dPAS isoform vs. pPAS isoform) between SH-SY5Y cells and HEK293T cells. Each dot is a gene with two selected 3′UTR isoforms (dPAS and pPAS isoforms). The top 20% (red) and bottom 20% (blue) genes, 932 each, based on ΔSS are highlighted in red and blue, respectively. **(G)** UCSC Genome Browser tracks of an example gene, *MRPL2*, whose dPAS isoform is relatively more stable than pPAS isoform in SH-SY5Y cells as compared to HEK293T cells. **(H)** As in G, except that data for the gene *NUP98* is shown, whose dPAS isoform is relatively less stable than pPAS isoform in SH-S5Y cells as compared to HEK293T cells. **(I)** Enriched motifs in aUTRs for red genes and blue genes shown in **(F)**. Motifs are represented by sequence logos based on enriched hexamers in each set.

We next wanted to identify transcript features that made RNA stability control in SH-SY5Y cells distinct as compared to other cells. Using a regression model, we examined various transcript features that correlated with transcript SS differences (ΔSS) between SH-SY5Y cells and HEK293T cells. The features we used included exonic and intronic sizes, splicing parameters, nucleotide contents of different regions, etc. (see Section 4 for detail). We found that GC and U contents were the top two features correlated to ΔSS (Figure 4A). Interestingly, whereas GC contents of the whole gene and of the last exon positively contributed to ΔSS, U contents of these regions negatively contributed to ΔSS (Figure 4A). In addition, 3′UTR size and number of PASs in 3′UTRs also negatively impacted ΔSS, albeit to a much lesser extent (Figure 4A). The effect of 3′UTR GC content on transcript stability difference between SH-SY5Y and HEK293 cells could also be demonstrated by the significant difference in GC content between the transcripts that were more stable in SH-SY5Y (top 20% of ΔSS) and those that were more stable in HEK293T (bottom 20% of ΔSS) (*p* < 2.2 × 10^−16^, K-S test, Figure 4B). Importantly, transcripts with high GC contents (top 20%) were more likely to be upregulated in neurogenesis than transcripts with low GC contents (bottom 20%, Figure 4C), highlighting the potential functional relevance of GC content for gene expression in neuronal cells.

Because RNAs with higher GC contents could adopt more stable secondary structures than those with lower GC contents (Zheng et al., 2020), we next set out to examine how RNA structures might be related to the RNA stability difference between SH-SY5Y and HEK293T cells. We first calculated the medium minimum folding energy of all sub-sequences of a given 3′UTR (100 nt each, see Materials and Methods for detail). We found that transcripts that were more stable in SH-SY5Y cells (top 20% ΔSS, SH-SY5Y vs. HEK293T) had significantly lower MFE values than those that were more stable in HEK293T cells (bottom 20% ΔSS, SH-SY5Y vs. HEK293T) (*p* < 2.2 × 10^−16^, K-S test, Figure 4D), supporting the notion that RNA structures positively contribute to ΔSS (SH-SY5Y vs. HEK293T).

To further corroborate RNA structure prediction results, we analyzed a dataset previously generated by Rouskin et al., which probed RNA structures *in vivo* using dimethyl sulfate treatment followed by sequencing (DMS-seq) (Rouskin et al., 2014). With DMS-Seq data, RNA structures are represented by Gini indices. A high Gini index indicates a high possibility of RNA secondary structures. Consistent with the MFE-based RNA structure prediction result, transcripts that were more stable in SH-SY5Y cells (top 20% ΔSS, SH-SY5Y vs. HEK293T) showed significantly higher DMS-seq Gini indices than those that were more stable in HEK293T cells (bottom 20% ΔSS, SH-SY5Y vs. HEK293T) (*p* < 2.2 × 10^−16^, K-S test, Figure 4E). Taken together, these results indicate that high GC contents and likely more stable RNA secondary structures may make transcripts more stable in SH-SY5Y cells compared to HEK293T cells.

We next asked whether the GC content-related RNA stability difference between SH-SY5Y and HEK293T cells could impact 3′UTR isoform stability differences in different cells. To this end, we first calculated 3′UTR isoform stability difference, or ΔSS, between dPAS isoform and pPAS isoform per gene in both SH-SY5Y and HEK293T cells (Figure 4F). While ΔSS values were generally correlated between the 2 cell types (*r* = 0.45, Pearson Correlation, Figure 4F), some differences were discernable. Based on ΔSS differences, we identified genes that had higher ΔSS values in SH-SY5Y cells (top 20%, red genes in Figure 4F) and genes that had higher ΔSS values in HEK293T cells (bottom 20%, blue genes in Figure 4F). Two example genes *MPPL2* and *NUP98* are shown in Figures 4G,H, respectively. Whereas *MPPL2* had a larger ΔSS value in SH-SY5Y cells than HEK293T cells (2.5 vs. −1.6), *NUP98* showed the opposite trend (−0.6 vs. −4.2). Interestingly, we found that GC-rich motifs were highly enriched in aUTRs of red genes (Figure 4I, left), whereas U-rich motifs were enriched in aUTRs of blue genes (Figure 4I, right, U shown as T). This result indicates that GC and U contents in aUTRs contribute to 3′UTR isoform stability variations between SH-SY5Y and HEK293T cells, corroborating our results based on transcript comparison across genes (Figures 4A–E). Interestingly, we also found that red and blue genes were enriched with GO terms related to mitochondrial functions (Supplementary Figure S4A) and cytosolic metabolic functions (Supplementary Figure S4B), respectively, suggesting that 3′UTR isoform difference in stability may have distinct functional consequences in the 2 cell types. Taken together, our results indicate that GC-rich motifs and likely RNA structures could make transcripts and isoforms more stable in SH-SY5Y cells than HEK293T cells.

### 2.6 Intronic Polyadenylation Isoforms are Generally Unstable

**FIGURE 5:**
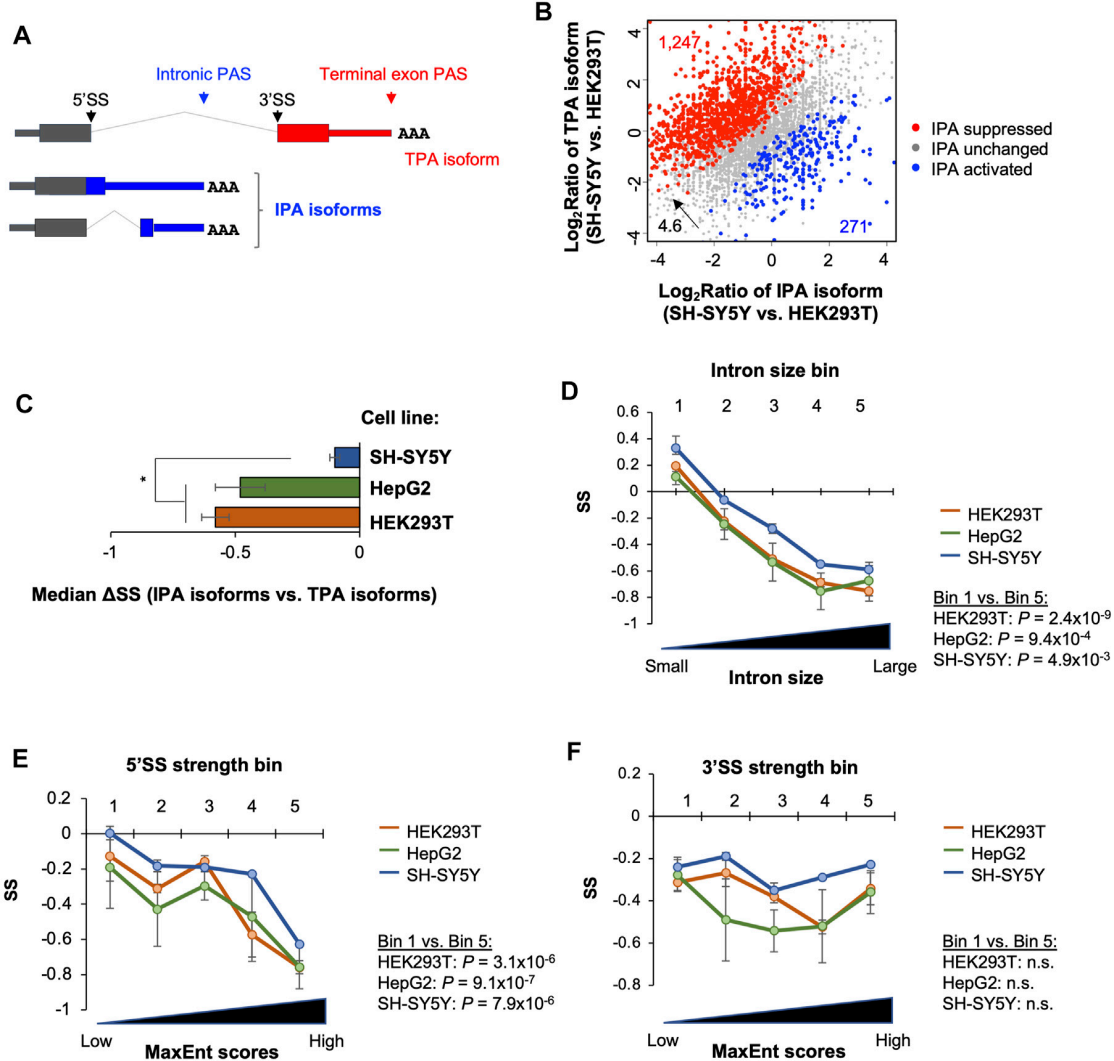
Stability analysis of intronic polyadenylation isoforms. **(A)** Schematic of intronic polyadenylation (IPA) isoforms and 3′-most terminal exon polyadenylation (TPA) isoform. **(B)** Comparison of IPA isoform stability with TPA isoform stability in three cell lines. **(C)** Median ΔSS between IPA and TPA isoforms in three cell lines. Significance is based on the Wilcoxon test comparing the cell lines. **(D)** Impact of intron size on IPA isoform stability. IPA isoforms are divided into five equally sized bins based on the size of intron containing the IPA site. P‐value (Wilcoxon test) for significance of difference between bin 1 and bin 5 in each cell line is indicated. **(E)** As in C, except that IPA isoforms are divided into five equally sized bins based on the 5′SS strength (MaxEnt score) of intron containing the IPA site. (F) As in C, except that IPA isoforms are divided into five equally sized bins based on the 3′SS strength (MaxEnt score) of intron containing the IPA site. n.s., not significant (*p* > 0.05, Wilcoxon test).

A sizable fraction of APA isoforms use PASs in introns (illustrated in Figure 5A) (Tian et al., 2007; Hoque et al., 2013; Singh et al., 2018). With our data, we identified 36,368 intronic polyadenylation (IPA) sites in 10,191 genes (Supplementary Figure S5A). Similar numbers of genes in the 3 cell lines expressed IPA isoforms (Supplementary Figure S5). By comparing transcript abundances of IPA isoforms versus 3′-most exon APA isoforms (called TPA isoforms for simplicity, illustrated in Figure 5A), we found that genes showing IPA suppression in SH-SY5Y versus HEK293T (red genes, Figure 5B) outnumbered those showing IPA activation in SH-SY5Y versus HEK293T (blue genes, Figure 5B) by 4.6-fold. This result indicates global IPA suppression in SH-SY5Y compared to HEK293T. Notably, IPA was also suppressed in NSC differentiation to neurons (Supplementary Figure S5B). These results are in good agreement with the notion that neuronal cells prefer to use PASs in the last exon (Zhang et al., 2005; Taliaferro et al., 2016).

We next compared transcript stability between IPA isoforms and TPA isoforms. We found that median ΔSS (IPA isoform vs. TPA isoforms) in all 3 cell lines were negative (Figure 5C), indicating that IPA isoforms in general were less stable than TPA isoforms. Interestingly, based on median ΔSS, IPA isoforms were significantly less unstable in SH-SY5Y cells compared to the two other cell lines (*p* < 0.05, *t*-test, Figure 5C). Therefore, for both 3′UTR APA isoforms (Figure 3) and IPA isoforms, stability difference between isoforms varies across cell lines, and, strikingly, the more stable isoforms in a cell also appeared to be less abundant at the steady state level as compared to other isoforms (see Section 3).

We next wanted to examine gene features related to IPA isoform stability as we did with 3′UTR isoforms. However, because our 3′READS + reads for IPA transcripts were largely located in the middle of an intron, we could not accurately derive sequence features, such as CDS size, 3′UTR size, etc., for most IPA isoforms. We therefore focused on the introns in which IPA sites were identified, including intron size, 5′ splice site (5′SS) strength, and 3′ splice site (3′SS) strength. Splice site strengths were based on the maximum entropy (MaxEnt) method (Yeo and Burge, 2004). We divided IPA site-containing introns into five equally sized bins based on each of these three features, and examined IPA isoform SSs across the bins. We found that IPA isoform stability descresed as intron size (Figure 5D) and 5′SS strength (Figure 5E) increased in all 3 cell lines. This trend, however, was not discernible with 3′SS strength (Figure 5F). Consistently, transcripts in the top 20% (bin 5) and bottom 20% (bin 1) groups based on intron size and 5′SS strength were significantly different in stability (*p* < 0.05, Wilcoxon test, Figures 5D,E) in all 3 cell types. In contrast, the difference was not significant based on 3′SS strength (Figure 5F). Together, these data indicate that IPA isoforms are generally unstable, and intron size and 5′SS strength are related to their stability control.

### 2.7 Distinct Stability of lncRNAs and Mitochondrial RNAs in SH-SY5Y Cells

**FIGURE 6:**
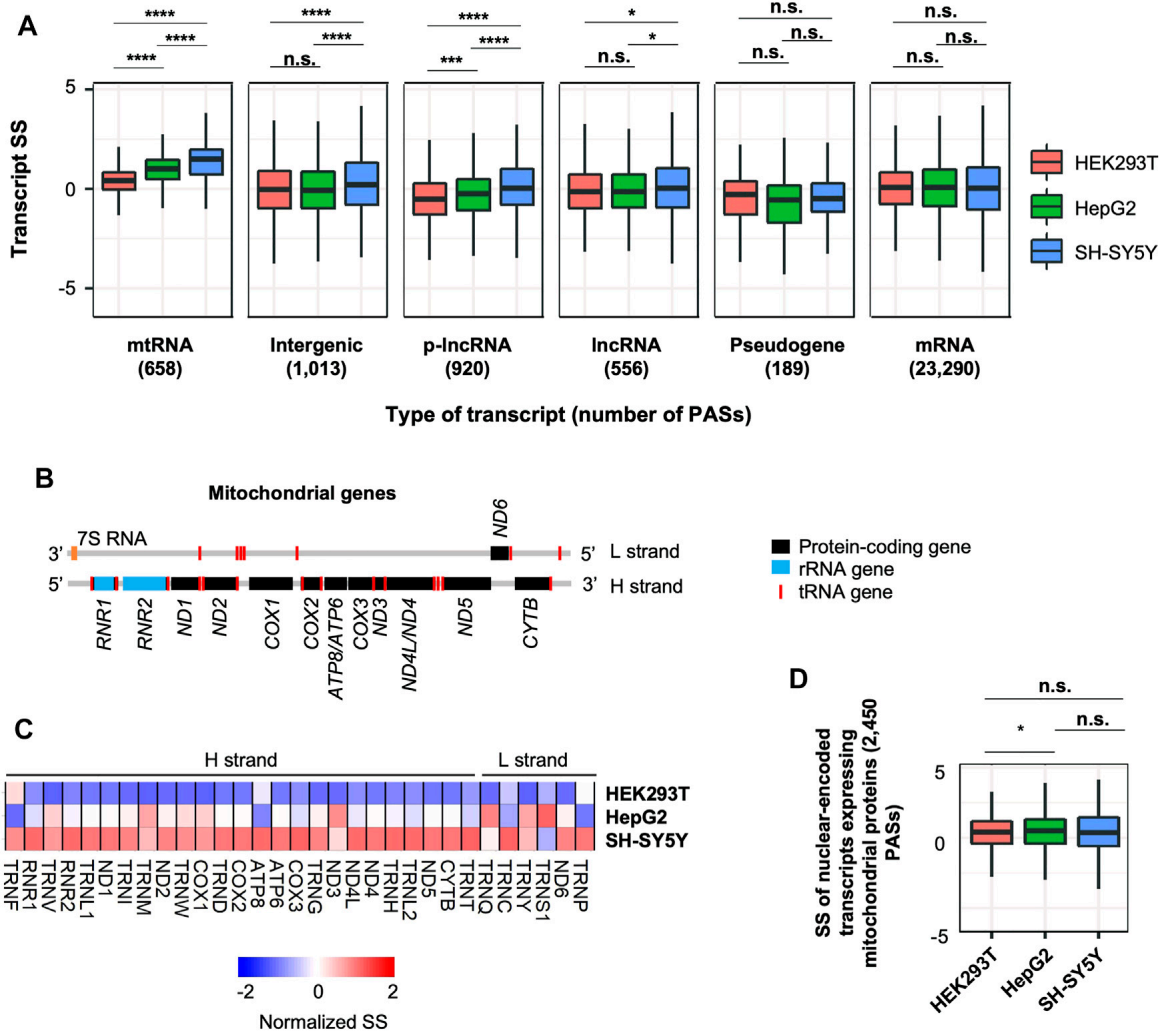
Distinct stability control of lncRNAs and mitochondrial RNAs in SH-SY5Y cells. **(A)** Boxplots showing SS of different RNA species in 3 cell lines. *p*-value (Wilcoxon test) for significance of difference between cell lines is indicated. Only the transcripts detected in all cell lines are included, and only the RNA species with >100 detected transcripts are shown. n.s., *p* > 0.05; *, *p* ≤ 0.05; **, *p* ≤ 0.01; ***, *p* ≤ 0.001; ****, *p* ≤ 0.0001. **(B)** Schematic of transcripts expressed from heavy (H) and light (L) strands of the mitochondrial genome. **(C)** Heatmap showing normalized SS of mitochondrial transcripts in 3 cell lines. **(D)** SS of nuclear-encoded transcripts that encode mitochondrial proteins in 3 cell lines.

In addition to protein-coding transcripts, our 3′READS + data identified many lncRNAs, including canonical lncRNAs, p-lncRNAs and eRNAs, RNAs encoded by the mitochondrial genome (named mtRNAs), pseudogene transcripts, and unannotated intergenic RNAs (Supplementary Figure S1C). We calculated SS of these RNAs and asked whether their stability controls were different among the three cell types (Figure 6A). Interestingly, p-lncRNAs and mtRNAs both showed discernable stability variations across the cell lines. They both were significantly more stable in SH-SY5Y cells than in HepG2 or HEK293T cells (*p* < 0.05, Wilcoxon test, Figure 6A). In contrast, mRNAs overall did not show significant differences between the cell lines (*p* > 0.05, Wilcoxon test, Figure 6A). In addition, pseudogene and intergenic transcripts overall showed comparable stability levels between the cell lines (Figure 6A).

We next further examined mtRNA genes, which included 13 protein-coding genes, 2 rRNA genes, and 22 tRNA genes (Figure 6B and Supplementary Figure S6A). We found that most of them were indeed more stable in SH-SY5Y cells compared to HEK293T and HepG2 cells (Figure 6C). Importantly, this trend was not detected in nuclear-encoded RNAs that encoded for mitochondrial proteins (Figure 6D), indicating that mitochondria in SH-SY5Y cells have a distinct RNA stability control than other 2 cell types. Notably, mtRNAs are highly expressed in brain tissues based on the Genotype-Tissue Expression (GTEx) data (GTEx Consortium et al., 2017) (Supplementary Figure S6B, see Section 4 for detail), suggesting that stability control may play a role in expression regulation of mitochondrial transcripts in neurons.

## 3 Discussion

In this study, we systematically studied poly(A)+ RNA stability in three human cell lines, namely, HEK293T, HepG2, and SH-SY5Y. We found that while mRNA stability controls are generally similar in these cells, certain transcripts display differential stability levels across the cell lines, especially in SH-SY5Y cells. We show that GC content and RNA secondary structures of transcripts correlate with distinct mRNA stability values in SH-SY5Y cells, which also impact 3′UTR isoform stability differences in these cells. Given the relevance of our SH-SY5Y versus HEK293T comparison to neurogenesis in gene expression and APA regulation, we conclude that modulation of mRNA stability could contribute to establishment of neuronal cell identity.

Our RNA stability analysis is based on comparison of transcript abundances in newly made versus pre-existing RNA pools. We show that the Stability Score (SS) derived from this comparison are well correlated with RNA decay rates that were measured by using transcriptional shutdown. As such, our method offers a simple and meaningful approach to examine RNA stability. On the other hand, it is worth noting that the accuracy of SS could be compromised if there are alternations of transcription during the course of metabolic labeling. In other words, when newly made and pre-existing RNAs are produced in distinct transcriptional contexts, our method would be affected by large noises. However, we do not expect this to be an issue when cells are under normal growth conditions, as in this study.

We show that GC content and RNA structures could impact stability differences between 3′UTR isoforms, corroborating the gene-to-gene analysis result. It is worth noting that APA isoforms that differ in 3′UTRs offer an accurate and efficient approach to examine the impact of 3′UTR on mRNA metabolism. This is because the 3′UTR isoforms presumably have identical sequences except for their aUTRs; their comparison, therefore, addresses potential, confounding features in CDS and 5′UTR. The underlying mechanism(s) for RNA stability control via GC content and RNA secondary structures, however, is unclear. There are a group of RNA-binding proteins that specifically interact with sequence motifs with high GC contents (Dominguez et al., 2018; Van Nostrand et al., 2020) and/or RNA structures (Tian et al., 2004; Li et al., 2010; Pan et al., 2018). For example, recent studies indicated G3BP1 and UPF1 in binding highly structured 3′UTRs, which leads to structure-mediated RNA decay (Imamachi et al., 2017; Fischer et al., 2020). Intriguingly, we recently found that GC content and RNA structures in 3′UTRs help mRNA associate with the endoplasmic reticulum (ER) (Cheng et al., 2021). How RNA stability interfaces with RBP interactions and ER association needs to be studied in the future.

Our finding that long 3′UTR isoforms are generally less stable than short 3′UTR isoforms is largely in line with our previous studies (Zheng et al., 2018; Cheng et al., 2020; Shin et al., 2021). Notably, an earlier study using transcriptional shutdown by Actinomycin D reported a similar but less prominent trend in NIH3T3 cells (Spies et al., 2013). The discrepancy could stem from differences in sequencing and analysis methods. For example, in the study by Spies et al. mRNA half-life was calculated over a period of time after transcriptional shutdown. In contrast, we generated SS based on newly made and pre-existing RNAs. While SS is sensitive in detecting stability differences, it does not measure half-life *per se*. Therefore, subtle differences in half-life values could be significant in this work but not in the Spies et al. study. Nevertheless, our comparative approach using multiple cell lines enabled us to identify cell type-specific differences in RNA stability, mitigating any intrinsic biases in using SS to measure stability.

We found that IPA isoforms are generally less stable than isoforms using 3′-most exon PASs. This result suggests that while some IPA isoforms could diversify the protein-coding potential of genes, most IPA events may lead to unstable RNAs that do not have substantial impacts on cell functions. In other words, IPA may be employed mainly to downregulate gene expression. Interestingly, we found that both intron size and 5′SS strength play negative roles in IPA isoform stability. 5′SS strength may be related to nuclear retention of IPA transcripts through U1 snRNP, as shown recently for lncRNAs (Yin et al., 2020). The negative impact of intron size on IPA isoform stability may be due to the possibility that large introns tend to give rise to IPA isoforms with long 3′UTRs, a negative feature for transcript stability. However, because our short read data do not cover full-length sequence, we could only infer these potential mechanisms at this point. Future work using long read sequencing would provide more definitive conclusions.

Our data indicate that isoform stability variation counters APA site choice, i.e., the isoforms preferentially expressed in 1 cell type are also less stable in this cell type, as compared to other cell types. Interestingly, this trend applies to both 3′UTR APA isoforms and IPA isoforms, indicating that stability regulation of a specific transcript is connected to its 3′ end processing activity. Notably, a recent study by the Struhl lab reported a similar compensatory link between transcript decay and its 3′ end processing in yeast (Moqtaderi et al., 2022). This phenomenon is reminiscent of mRNA degradation-mediated transcriptional buffering that was previously reported in multiple species (Haimovich et al., 2013; Sun et al., 2013). Whether XRN1, the protein implicated in the decay-transcription buffering (Sun et al., 2013), is also involved in the link between APA isoform decay and APA site choice awaits further experimentation.

Several types of lncRNAs, including regular lncRNAs and p-lncRNAs, were found to be generally more stable in SH-SY5Y cells compared to HEK293T or HepG2 cells. Regular lncRNAs are stand-alone genes, similar to mRNAs. p-lncRNAs are associated with gene promoters, also known as upstream antisense RNAs or PROMPTs (Preker et al., 2008; Hon et al., 2017). Both RNA species are enriched, if not exclusively localized, in nucleus. As such, their stability is under the control of nuclear exosome (Kilchert et al., 2016; Schmid and Jensen, 2019). Our result, therefore, appears to indicate that nuclear degradation machinery is less active in SH-SY5Y cells than the other 2 cell lines. Whether the increased stability of lncRNAs in SH-SY5Y cells would impact gene regulation in neurogenesis would be an interesting direction to explore (Liu et al., 2016).

Our finding that mitochondrial RNAs are more stable in SH-SY5Y cells than other 2 cell lines may have important implications for neurobiology. Mitochondrial dysfunctions are known to be involved in the pathogenesis of several neurodegenerative disorders (Federico et al., 2012; Bader and Winklhofer, 2020), highlighting the role of mitochondria in a high energy demanding environment like brain. Consistently, mitochondrial genes are highly expressed in neuronal tissues compared to non-neuronal tissues (Supplementary Figure S6B). How mitochondrial RNA stability regulation contributes to neuronal cell functions is an open question that needs to be addressed in the future.

## 4 Materials and Methods

### 4.1 Cell Culture and Neural Stem Cell Differentiation

Human HEK293T and HepG2 cells were cultured in high glucose DMEM with 10% fetal bovine serum (FBS). SH-SY5Y cells were cultured in DMEM/F12 with 10% FBS, 1% sodium pyruvate, and 1% glutamate. All media contained 1% Penicillin/Streptomycin solution (Sigma). Human neural stem cells (NSCs) were obtained from Vescovi et al. (Vescovi et al., 1999). NSCs were cultured in NS-A Basal medium. Differentiation of NSCs to neurons was carried out by following the protocol described in Vescovi et al. (Vescovi et al., 1999). Deidentified primary human NSC line was previously developed (Vescovi et al., 1999). For neuronal induction, individual spheres were mechanically dissociated and transferred at a density of 2.5 × 10^4^ cells/cm^2^ onto matrigel-coated chamber-slides in the presence of 20 ng/ml FGF2. After 72 h, cultures were shifted to NS-A Basal medium containing 2% FBS and were grown for another 2 weeks. Neurons were harvested by using accutane to dissociate cells, and RNA was extracted by using Trizol.

### 4.2 Metabolic Labeling of RNA and RNA Isolation

Cells at ∼70% confluency were used for metabolic labeling of RNA as previously described (Zheng et al., 2018). Briefly, cell media were supplemented with 50 μM of 4-thiouridine (4sU, Sigma) for 1 h before cell harvest. Total RNA was extracted by using TRIzol (Thermo Fisher Scientific). Newly made (4sU-labeled) and pre-existing RNA pools were fractionated by using a previously described protocol (Radle et al., 2013). Briefly, 100 μg of total RNA was biotinylated with 200 μg of biotin-HPDP (Thermo Fisher Scientific), and the biotinylated RNA was captured by Dynabeads MyOne Streptavidin C1 (Thermo Fisher Scientific). The unbound, flow-through (FT) RNA was collected after extensive washing of the beads. Biotinylated RNA (4sU) was eluted from the beads by using DTT.

### 4.3 3′READS+

The 3′READS+ procedure was carried out as previously described (Zheng et al., 2016). Briefly, poly(A)+ RNA was captured by using oligo (dT)_25_ magnetic beads (NEB) and was fragmented on-bead by RNase III (NEB). After washing away free RNA fragments, poly(A)+ RNA fragments were eluted from the beads and precipitated with ethanol, followed by ligation to heat-denatured 5′ adapter (5′-CCUUGGCACCCGAGAAUUCCANNNN) with T4 RNA ligase 1 (NEB). The ligated products were captured by biotin-T_15_-(+TT)_5_ (Exiqon, and +T indicates locked nucleic acid) bound to Dynabeads MyOne Streptavidin C1 (Thermo Fisher Scientific). After washing, RNA fragments on the beads were digested with RNase H and then eluted from the beads. After precipitation with ethanol, RNA fragments were ligated to a 5′ adenylated 3′ adapter (5′-rApp/NNNGATCGTCGGACTGTAGAACTCTGAAC/3ddC (Bioo Scientific) with T4 RNA ligase 2 (truncated KQ version, NEB). The ligation products were then reverse transcribed by using M-MLV reverse transcriptase (Promega), followed by PCR amplification with Phusion high-fidelity DNA polymerase (NEB) and bar-coded PCR primers for 12–18 cycles. PCR products were size selected twice with AMPure XP beads (Beckman Coulter). The size and quantity of the cDNA libraries were examined on an Agilent Bioanalyzer and sequenced on an Illumina HiSeq machine (1 × 150 bases).

### 4.4 3′READS+ Data Analysis

3′READS+ data were analyzed as previously described (Hoque et al., 2013; Zheng and Tian, 2017; Zheng et al., 2018). The 5′ adapter was removed using Cutadapt (Martin, 2011) and reads with <23 nt were discarded. The retained reads were mapped to the human genome (hg19) using bowtie2 local mode (Langmead and Salzberg, 2012). The six random nucleotides at the 5′ end (from the 3′ adapter) were removed using the setting “-5 6” in bowtie2. Reads with a mapping quality score ≥10 were kept for further analysis. Reads with two or more non-genomic 5′Ts after alignment were called PAS reads. Cleavage sites were clustered within 24 nt (Hoque et al., 2013) and were assigned to genes based on RefSeq (release 83) annotations (Pruitt et al., 2007). RNA-seq data-supported 3′ end extension was applied to improve 3′ end region annotation as previously described (Wang et al., 2018b). Genic PASs were annotated by using the RefSeq with the largest genomic span. Human lncRNA annotations were based on data from the FANTOM5 database (Hon et al., 2017). PAS reads mapped to genes were normalized by the median ratio method in DESeq (Anders and Huber, 2010). Only the isoforms with read count greater than five in at least one of the samples were used. PASs on chromosome M were annotated to mitochondrial genes by using MitoCarta2.0 (Calvo et al., 2016).

### 4.5 Stability Analysis of Transcripts

For each transcript with a defined PAS, its abundances (reads per million mapped, or RPM) in flow-through (FT) sample and 4sU sample were calculated and normalized by using the DESeq method. The log_2_ (RPM of FT sample/RPM of 4sU sample) value is called Stability Score (SS). Two biological replicates were averaged. Scaled mRNA decay rate data was obtained from a previous study (Wu et al., 2019), which involved decay rate calculation after transcriptional shutdown by Actinomycin D. Differential stability analysis across cell lines was carried out by using the ANOVA test.

### 4.6 APA Isoform Analysis

For 3′UTR isoform analysis, the two APA isoforms containing 3′-most exon PASs with the highest expression levels were selected**.** Differential expression of proximal PAS and distal PAS isoforms was carried out by using DEXSeq (Anders et al., 2012). Significant events were those with *p* < 0.05 (Fisher’s exact test or DEXSeq analysis) and relative abundance difference >5%. Relative expression (RE) of the two isoforms was calculated by log_2_ (distal PAS isoform RPM/proximal PAS isoform RPM). aUTR size was the distance between the proximal and distal PASs in the 3′UTR.

### 4.7 Gene Ontology Analysis

Gene ontology analysis was carried out by using the GOstats package in R (Falcon and Gentleman, 2007). The Fisher’s exact test was used to calculate *p*-values to indicate significance of association between a gene set and a GO term. GO terms associated with more than 1,000 genes were considered too generic and were discarded. To reduce redundancy, any GO term that overlapped with a more significant term by >90% was removed.

### 4.8 Gene Feature Analysis

Gene features were based on RefSeq annotations. The Pearson correlation *r* value and individual and cumulative R^2^-value were calculated using the “cor” and “lm” functions in R. The PAS count of a gene and conservation of PAS were obtained from PolyA_DB v3 (Wang et al., 2018a). For intronic features, intron size was based on the RefSeq database, considering all RefSeq-supported splicing isoforms. The strengths of 5′ and 3′ splicing sites were calculated by using the MaxEntScan program (Yeo and Burge, 2004).

### 4.9 Sequence Motif Analysis

Nucleotide content in a specific region was calculated by using the BSGenome package in R. Hexamer frequencies in 3′UTRs were calculated by using the Biostrings package in R and were compared between gene sets. *p*-values indicating significance of hexamer enrichment or depletion were based on the Fisher’s exact test. Sequence motifs were generated by using the Weblogo program (https://weblogo.berkeley.edu/).

### 4.10 RNA Structure Analysis

The RNAfold function of the ViennaRNA package (Hofacker and Stadler, 2006) was used to calculate Minimum Folding Energy (MFE) of folded RNA sequences. Each 3′UTR sequence was divided into a series of 100-nt sub-sequences with a 50-nt overlap between adjacent ones. The median MFE value all sub-sequences was used to represent the whole sequence. DMS-Seq data based on K562 cells (Rouskin et al., 2014) were downloaded from NCBI GEO (GSE4580). Gini indices were calculated as previously described (Rouskin et al., 2014). The median Gini Index of each 3′UTR was used for analysis.

## Data Availability

The datasets presented in this study can be found in NCBI GEO database (GSE189899).
