# Supplementary material for: Neuronal Cells Display Distinct Stability Controls of Alternative Polyadenylation mRNA Isoforms, Long Non-Coding RNAs, and Mitochondrial RNAs
Source: Front Genet. 2022 May 18;13:840369. doi: 10.3389/fgene.2022.840369 (PMC9159357; doi:10.3389/fgene.2022.840369)
Supplement: Supplementary file 1 [file DataSheet1.zip › Suppl Table 1 & 3.DOCX]

**Supplemental Tables**

**Table S1. Genes and PASs detected in different samples.**

| **Sample** | **No. of genes** | **No. of PASs** |
| --- | --- | --- |
| HEK293T, FT, replicate 1 | 15,904 | 67,460 |
| HEK293T, FT, replicate 2 | 16,019 | 68,998 |
| HEK293T, 4sU, replicate 1 | 16,591 | 80,063 |
| HEK293T, 4sU, replicate 2 | 17,167 | 90,854 |
| HepG2, FT, replicate 1 | 15,815 | 69,160 |
| HepG2, FT, replicate 2 | 15,307 | 66,189 |
| HepG2, 4sU, replicate 1 | 15,818 | 71,841 |
| HepG2, 4sU, replicate 2 | 15,550 | 74,631 |
| SH-SY5Y, FT, replicate 1 | 16,621 | 70,058 |
| SH-SY5Y, FT, replicate 2 | 16,307 | 64,042 |
| SH-SY5Y, 4sU, replicate 1 | 16,915 | 80,384 |
| SH-SY5Y, 4sU, replicate 2 | 16,766 | 76,948 |

**Table S3. Top GO terms (Biological Process) enriched for transcripts that are stable or unstable in all three cell lines.**

|  | | **GO Term** | **P-value** |
| --- | --- | --- | --- |
| **Stable Transcripts** | small molecule metabolic process | | 9.4E-16 |
|  | oxidation-reduction process | | 1.0E-11 |
|  | regulated exocytosis | | 3.3E-11 |
|  | single-organism carbohydrate metabolic process | | 6.8E-08 |
|  | ion transport | | 9.1E-08 |
|  | NADH metabolic process | | 3.3E-07 |
| **Unstable transcripts** | transcription from RNA polymerase II promoter | | 1.6E-13 |
|  | negative regulation of macromolecule biosynthetic process | | 2.3E-08 |
|  | atrioventricular valve morphogenesis | | 1.5E-05 |
|  | fat cell differentiation | | 4.6E-05 |
|  | regulation of hemopoiesis | | 1.1E-04 |
|  | axis specification | | 2.6E-04 |
