# Supplementary material for: Neuronal Cells Display Distinct Stability Controls of Alternative Polyadenylation mRNA Isoforms, Long Non-Coding RNAs, and Mitochondrial RNAs
Source: Front Genet. 2022 May 18;13:840369. doi: 10.3389/fgene.2022.840369 (PMC9159357; doi:10.3389/fgene.2022.840369)
Supplement: Supplementary file 1 [file DataSheet1.zip › Suppl. Figures.PDF]

Figure S1

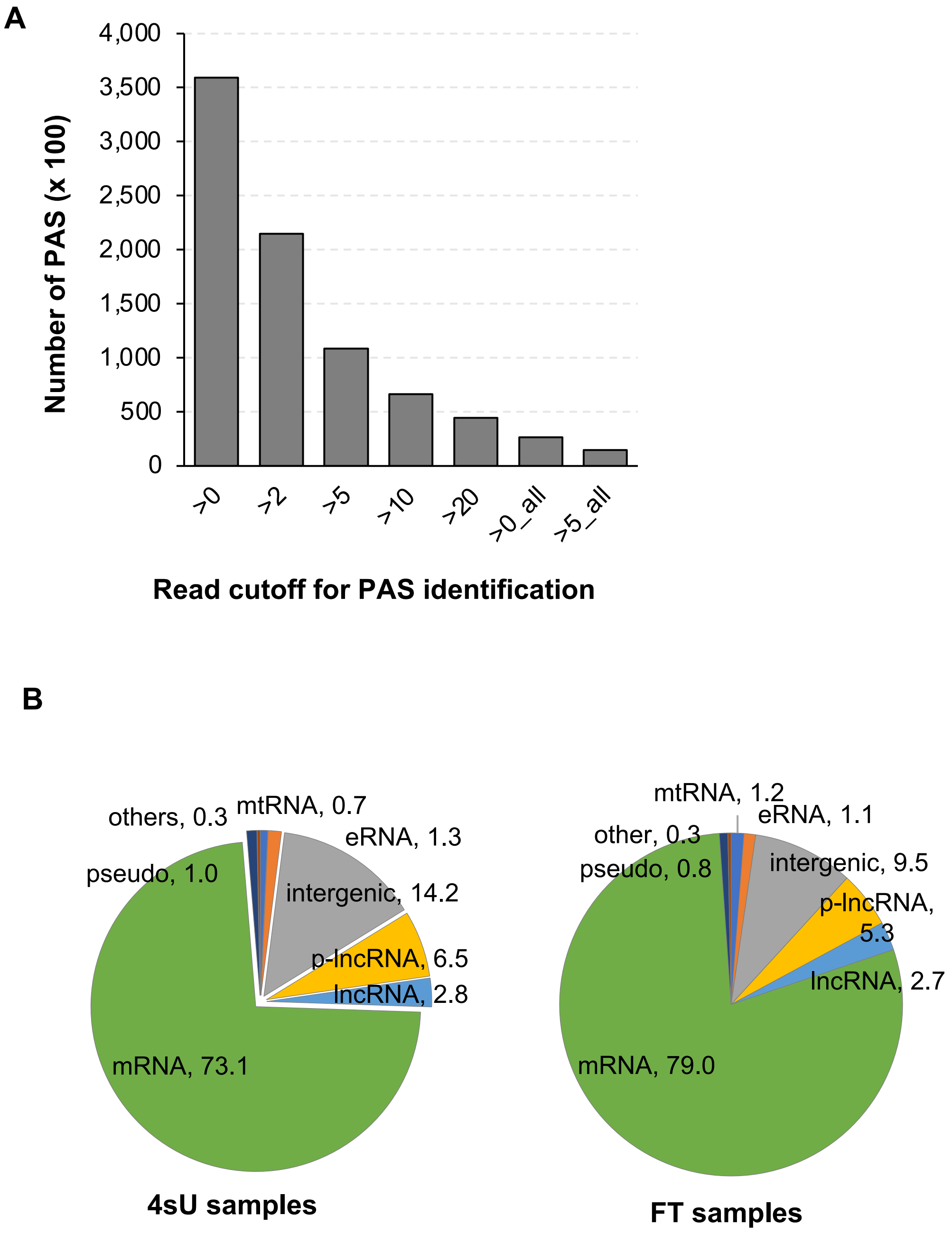

Number of transcripts

	4sU	FT
mtRNA	791	810
eRNA	737	1505
Intergenic	6,192	16,506
p-lncRNA	3,443	7,520
lncRNA	1,788	3,190
mRNA	51,479	84,169
pseudo	519	1,109
other	201	314

Figure S1. Sequencing data statistics.
A. Bar plot showing number of PASs based on different read cut-offs. >0, >2, >5, >10, and >20 correspond to number of reads per PAS in any of the samples; >0_all & >5_all correspond to number of reads per PAS in all samples.
B. Pie charts showing PAS distribution percentage for different RNA species in 4sU and FT samples. Data from all cells were combined .

Figure S2

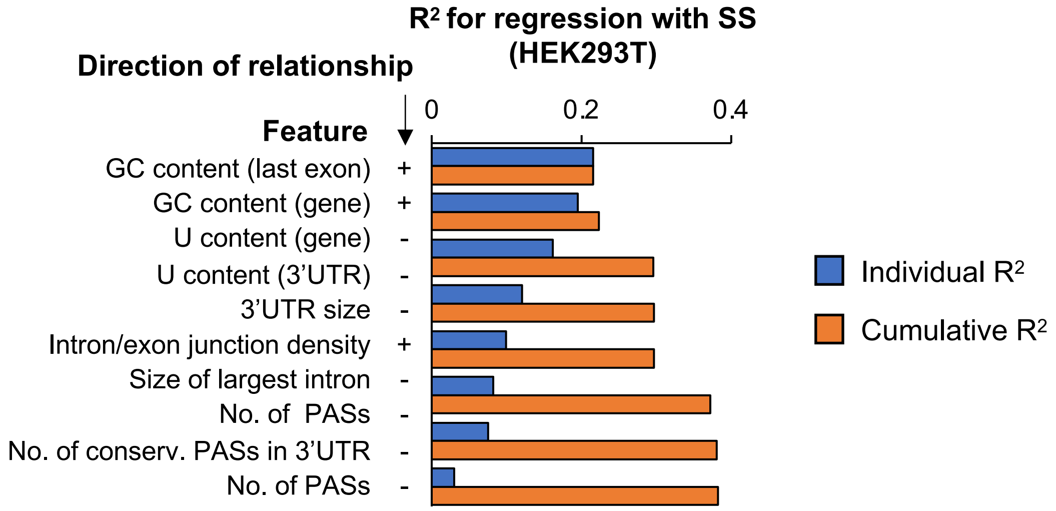

Figure S2. Top gene features correlated with Stability Scores (SS) in HEK293T cells.

Figure S3

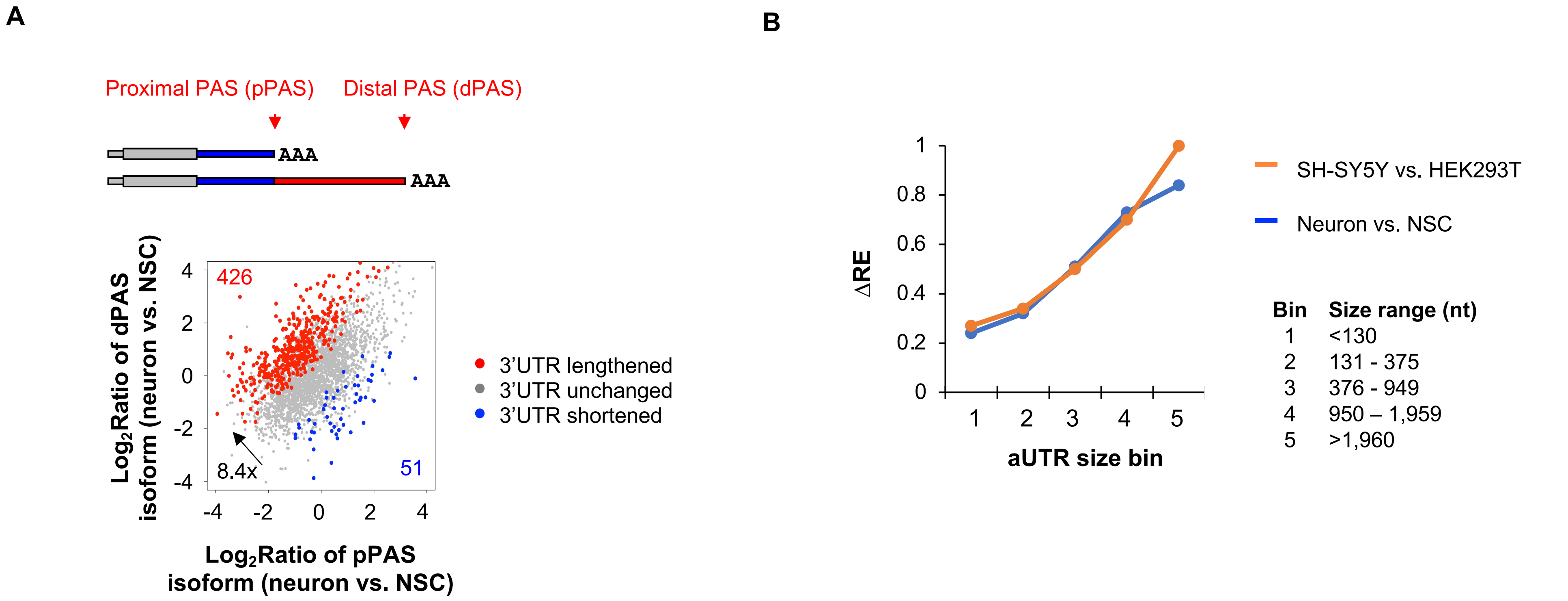

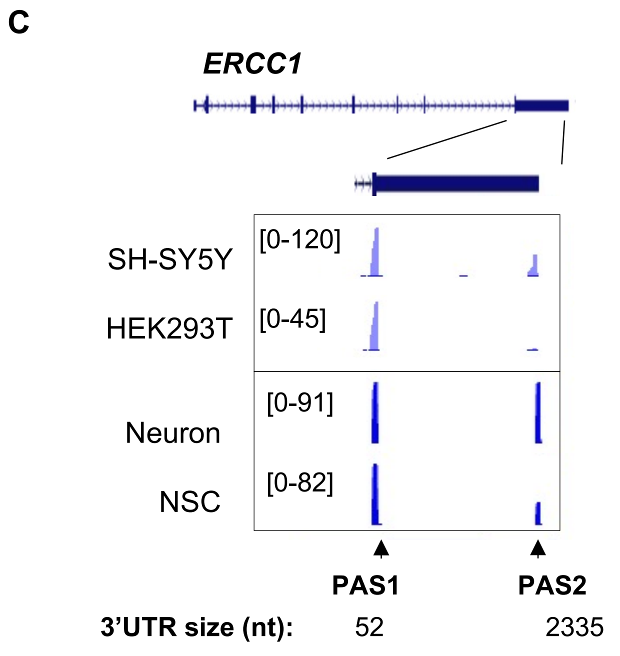

	RED
SH-SY5Y vs. HEK293T	2.75
Neuron vs. NSC	1.48

Figure S3. 3'UTR APA profile in SH-SY5Y confirms its neuronal identity
A. 3'UTR APA difference between differentiated neurons and NSCs.
B. aUTR size-dependent APA regulation in SH-SY5Y vs. HEK293T and neurons vs. NSCs.
C. UCSC Genome Browser tracks of an example gene *ERCC*, whose long 3'UTR isoform is more abundant in SH-SY5Y cells and differentiated neurons compared to HEK293T cells and NSCs, respectively.

Figure S4

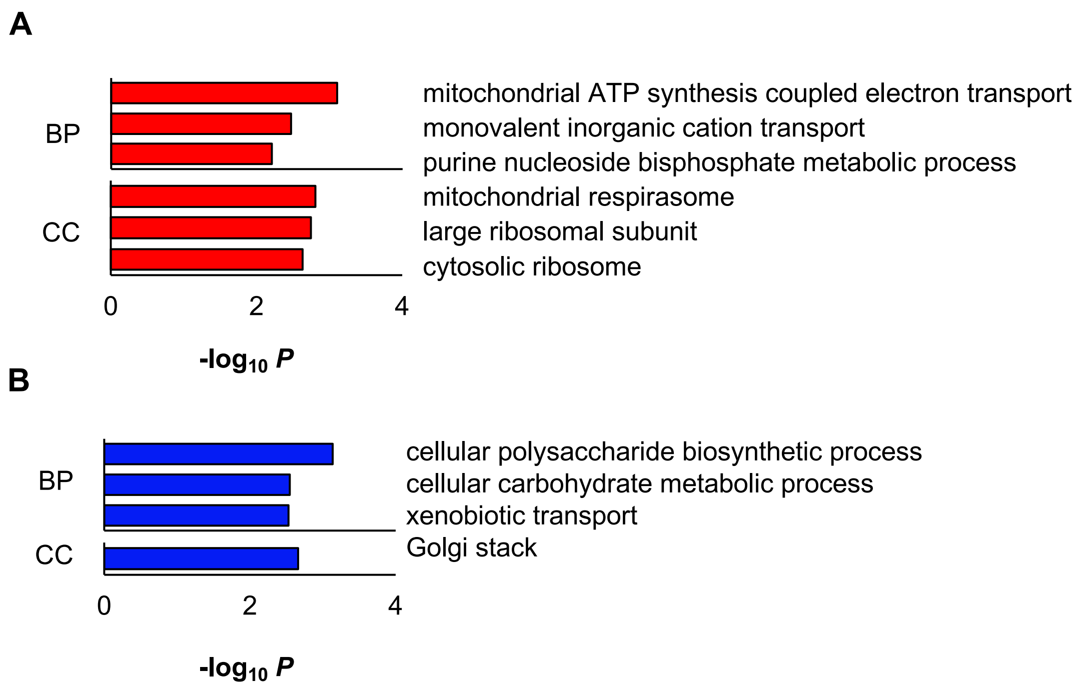

Figure S4. GO terms enriched for genes whose isoforms stability difference varies between SH-SY5Y and HEK293T cells.

A. Top GO terms (Biological Process and Cellular Component) enriched for red genes.

B. Top GO terms (Biological Process and Cellular Component) enriched for blue genes.

Figure S5

A

	Genes containing IPA sites (no. of reads per gene >0)	Genes containing IPA sites (no. of reads per gene >5)
HEK293T	8,971	4,690
HepG2	8,420	4,233
SH-SY5Y	7,338	3,284

B

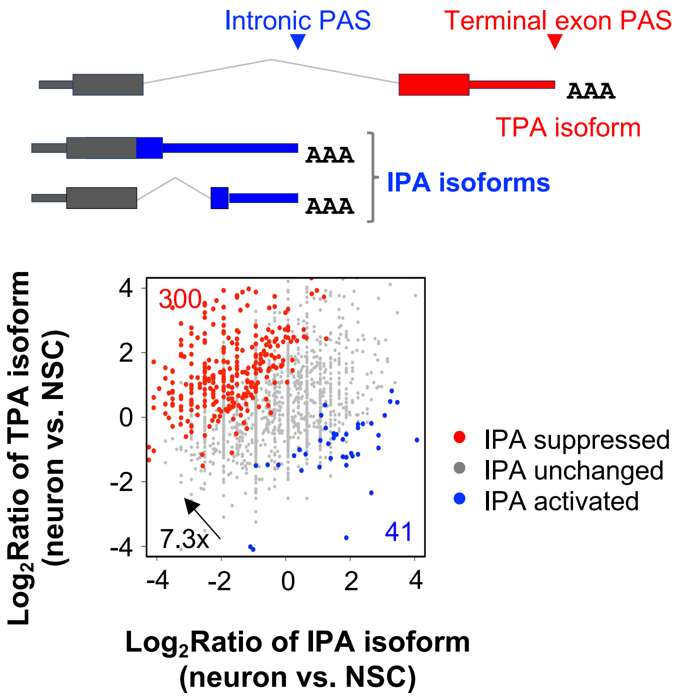

Figure S5. IPA isoforms detected in this study.
A. IPA isoforms detected in this study.
B. IPA isoform regulation in Neuron vs. NSC. IPA isoform expression change was compared to TPA isoform expression change.

Figure S6

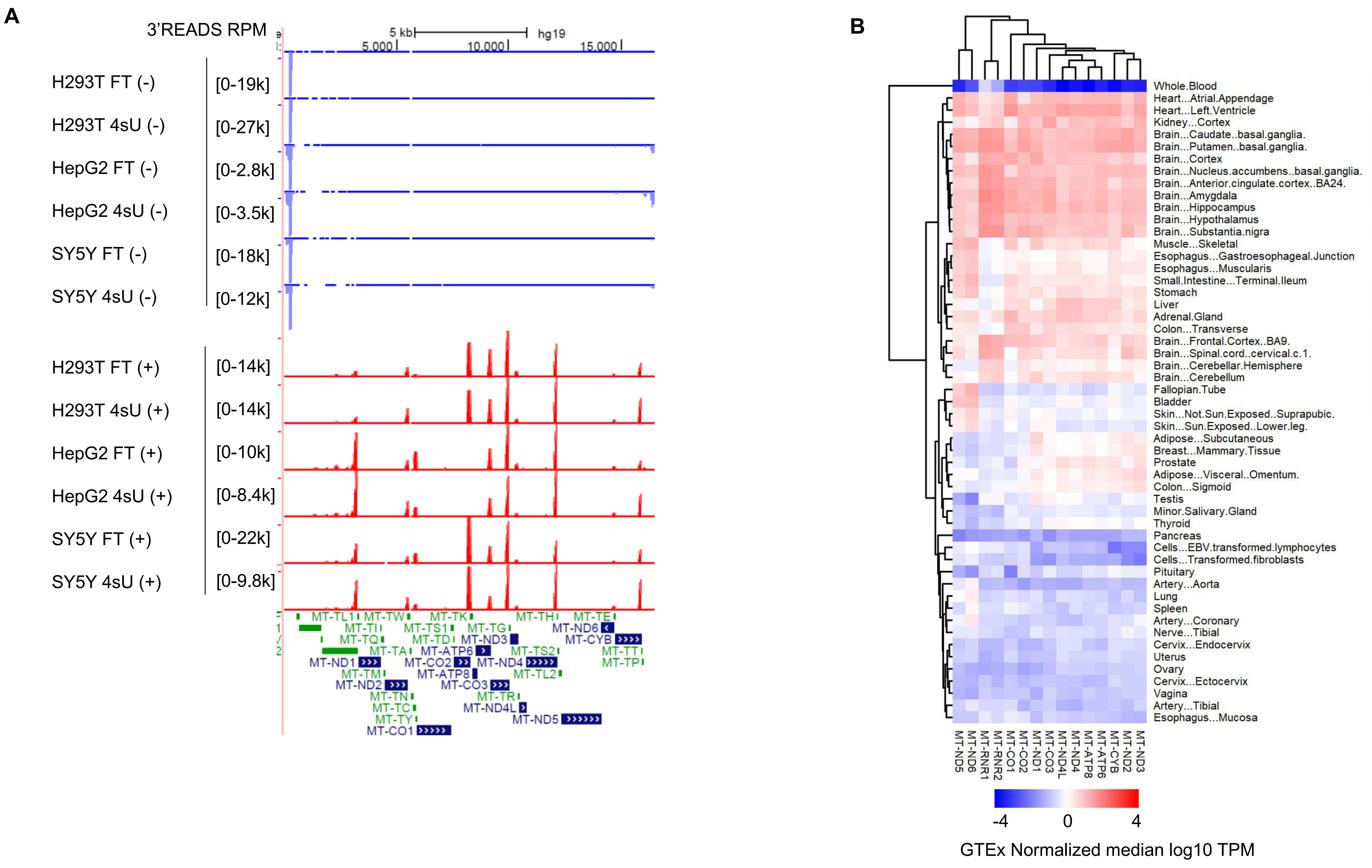

Figure S6. Mitochondrial RNA expression.

A. 3'READS+ data in this study.

B. GTEx data for selected mitochondrial RNAs (protein-coding RNAs and rRNAs only).
